# Exploring Fatigue Effects on Performance Variation of Intensive Brain–Computer Interface Practice

**DOI:** 10.3389/fnins.2021.773790

**Published:** 2021-12-02

**Authors:** Songwei Li, Junyi Duan, Yu Sun, Xinjun Sheng, Xiangyang Zhu, Jianjun Meng

**Affiliations:** ^1^State Key Laboratory of Mechanical Systems and Vibrations, Institute of Robotics, Shanghai Jiao Tong University, Shanghai, China; ^2^Key Laboratory for Biomedical Engineering of Ministry of Education of China, Department of Biomedical Engineering, Zhejiang University, Hangzhou, China

**Keywords:** brain–computer interface, motor imagery, fatigue, electroencephalogram (EEG), rest condition

## Abstract

Motor imagery (MI) is an endogenous mental process and is commonly used as an electroencephalogram (EEG)-based brain–computer interface (BCI) strategy. Previous studies of P300 and MI-based (without online feedback) BCI have shown that mental states like fatigue can negatively affect participants’ EEG signatures. However, exogenous stimuli cause visual fatigue, which might have a different mechanism than endogenous tasks do. Furthermore, subjects could adjust themselves if online feedback is provided. In this sense, it is still unclear how fatigue affects online MI-based BCI performance. With this question, 12 healthy subjects are recruited to investigate this issue, and an MI-based online BCI experiment is performed for four sessions on different days. The first session is for training, and the other three sessions differ in rest condition and duration—no rest, 16-min eyes-open rest, and 16-min eyes-closed rest—arranged in a pseudo-random order. Multidimensional fatigue inventory (MFI) and short stress state questionnaire (SSSQ) reveal that general fatigue, mental fatigue, and distress have increased, while engagement has decreased significantly within certain sessions. However, the BCI performances, including percent valid correct (PVC) and information transfer rate (ITR), show no significant change across 400 trials. The results suggest that although the repetitive MI task has affected subjects’ mental states, their BCI performances and feature separability within a session are not affected by the task significantly. Further electrophysiological analysis reveals that the alpha-band power in the sensorimotor area has an increasing tendency, while event-related desynchronization (ERD) modulation level has a decreasing trend. During the rest time, no physiological difference has been found in the eyes-open rest condition; on the contrary, the alpha-band power increase and subsequent decrease appear in the eyes-closed rest condition. In summary, this experiment shows evidence that mental states can change dramatically in the intensive MI-BCI practice, but BCI performances could be maintained.

## Introduction

Motor imagery (MI)-based brain–computer interface (BCI) provides a novel communication method by decoding a human’s motor intention from brain signals such as electroencephalogram (EEG) (He et al., 2020). Among various neural signatures, the endogenous oscillations from the sensorimotor cortex, i.e., sensorimotor rhythms (SMRs), are often used to decode MI (Wolpaw and Wolpaw, 2012). Currently, SMR-based MI-BCI enables the control of a robotic arm (Meng et al., 2016), virtual helicopters (Royer et al., 2010), communication (Perdikis et al., 2014), and video games (Bonnet et al., 2013). However, the usage of MI-BCI outside laboratories is currently still limited since many factors affect the performance of MI-BCI, such as the psychological state (Nijboer et al., 2010) and training effect (Meng and He, 2019).

Among various factors, fatigue effects during prolonged BCI operation are usually recognized to be negative factors (e.g., in P300-BCI) (Käthner et al., 2014). Another non-BCI study has shown that one session of MI training does not induce neuromuscular fatigue (Rozand et al., 2014). This could be generally considered as follows: EEG is a highly non-stationary signal, and a dramatic variation of features would be detrimental to the performance of BCI during prolonged operation. According to the research of Simon (Simon et al., 2011) and Seo (Seo et al., 2019), the power density of the alpha band increases with the degree of fatigue. And previous magnetoencephalography studies have also shown that physical fatigue (Tanaka and Watanabe, 2011) may influence the beta-band (13–30 Hz) activity during the operation of MI-BCI. The SMRs include both alpha and beta rhythms, which indicates that fatigue may impact the MI-BCI performance by influencing those rhythms. Furthermore, previous studies have shown that fatigue and other mental state changes may negatively affect BCI performance (Nijboer et al., 2010; Myrden and Chau, 2015). But few studies focus on the fatigue effects induced by MI-BCI operation itself. To our knowledge, only Talukdar (Talukdar et al., 2019) reports that the alpha power increases and the MI feature separability decreases during the prolonged MI tasks. Note that the common spatial pattern (CSP) was used to extract features in Talukdar’s offline analysis. However, without the online behavioral performances, the results of Talukdar and his colleagues can only reveal the fatigue effects on offline feature separability of MI-BCI. Thus, the fatigue effects on the MI-BCI with feedback require further investigations.

Rest is usually considered to be a solution to relieve fatigue in BCI and other applications. But the effects and efficiencies of different rest conditions are not clear in MI-BCI application. An early study (Marx et al., 2004) uses functional MRI to prove that patterns of associated brain activations during eyes-open and eyes-closed rest in darkness are different. Another EEG study (Boytsova and Danko, 2010) also reveals that the rest conditions differ in the variation of band power in EEG signals. These studies imply that different rest conditions might influence the efficiencies of rest differently.

This work aims to investigate the fatigue effects on MI-BCI with feedback, the impacts of rest conditions on the BCI performances (if fatigue effects exist), and electrophysiological indicators during prolonged MI-BCI operation. With the questions in mind, we hypothesize that BCI performance and electrophysiological indicators would change after a prolonged MI-BCI process due to fatigue; furthermore, rest conditions might alter the fatigue process significantly and subsequently affect the BCI performance and electrophysiological indicators. We designed protocols based on the hypothesis and assume that if the subjectively reported mental states (especially fatigue) change after a session of intensive BCI operation, then the operation time is considered long enough to induce fatigue. A randomized complete block design is utilized to contrast the rest conditions and restrict the learning effect on an MI-based online BCI with 400 trials of intensive training tasks.

## Materials and Methods

### Data Acquisition

#### Subjects

Fourteen healthy right-handed subjects participated in this study. Two subjects were unable to achieve acceptable performance (60% PVC) in the first session; thus, they were excluded from the experiment. The remaining 12 subjects finished all four sessions of experiments. The average age of the subjects was 22.2 ± 1.9 (mean ± SD) years. All of them were required to have a good rest and avoid caffeine consumption 1 day before the experiment. There were six BCI-naïve subjects in this study, and others have limited experience with MI-BCI experiments. The Institutional Review Board of Shanghai Jiao Tong University approved all procedures and protocols. Written informed consent was required for participation in the experiment.

#### Electroencephalogram Acquisition

The experiment was conducted in an electromagnetically shielded room. Subjects sat in front of a 24.5-inch LCD monitor at a distance of ∼70 cm. The EEG signals were measured with a 64-channel (63 channels exclude earlobe) g.HIamp (g.tec, Schiedlberg, Austria) system at a sampling rate of 1,200 Hz and band-pass filtered from 0.1 to 100 Hz. The electrodes on the left earlobe and forehead [AFz in extended 10–20 system (Nuwer et al., 1998)] were used as reference and ground, respectively. The impedances of all the active electrodes (g.SCARABEO) were kept below 30 kΩ. A notch filter of 50 Hz was applied to the raw EEG signals.

#### Questionnaire

Multidimensional fatigue inventory (MFI) (Smets et al., 1995) and short stress state questionnaire (SSSQ) (Helton, 2004) were used to measure subjects’ fatigue levels. The MFI contained 20 items covering general fatigue, physical fatigue, mental fatigue, reduced motivation, and reduced activity, aiming to grade the fatigue level in different types. As a supplement of MFI-20, the SSSQ was used for the assessment of task-induced subjective feelings. The SSSQ was a 24-item questionnaire differentiating stress into three aspects—engagement, distress, and worry—which revealed the change of emotional condition across the experiment. Each item was graded into five levels: totally inconsistent, slightly inconsistent, unsure, slightly consistent, and totally consistent. The two questionnaires were combined together and stored in an Excel sheet. Each subject was required to complete the self-reported questionnaires before and instantaneously after the experiment. Because all the subjects were native Chinese speakers, the questions were translated into Chinese by a well-trained bilingual (the questions were given in Supplementary Data Sheet 1).

### Experimental Setup

**FIGURE 1:**
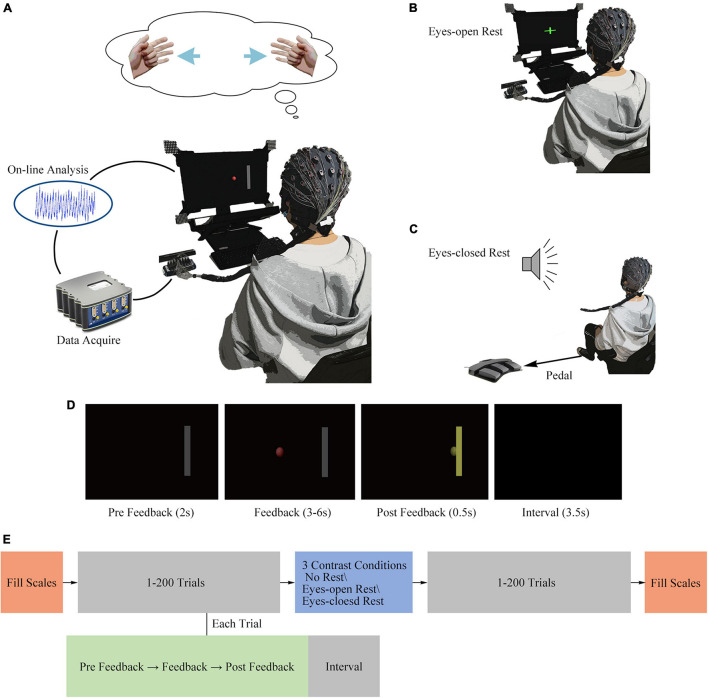
Experimental design. **(A)** Illustration of a subject controlling a cursor by imagining the movement of hands. **(B)** Illustration of a subject staring on the green cross in the center of the screen in the eyes-open rest condition. **(C)** Illustration of a subject stepping on the pedal according to an audio stimulus in the eyes-closed rest condition. **(D)** Screenshots of four different stages in a single trial. **(E)** Illustration of a session and a trial structure.

In order to contrast the different rest conditions, each subject was required to finish four sessions of the experiment, including one training session and three experimental sessions with various resting ways: no rest, 16-min eyes-open rest, and 16-min eyes-closed rest. In the training session, subjects were instructed to be familiarized with the MI-BCI task. Learning effect would inevitably emerge within and across sessions, which might interact with the factor of fatigue. Since learning effect was not a focus of this study, we would like to suppress the confounding factor of learning substantially. It is reported that subjects have significant improvements (R-square values increase) during the first few sessions of BCI operation (Meng and He, 2019). Thus, the factor of learning process could be restricted to the first session (400 trials, more than the previous study) to a certain degree. Furthermore, each subject finished three experimental sessions on different days, and the resting approach was arranged in a randomized complete block design regarding the rest conditions. Three rest conditions yielded six possible orders; the randomized complete block design went through the six orders twice (12 subjects), further restricting the possible confounding effect of learning. An experimental scenario is illustrated in Figure 1A.

The session and trial structures are shown in Figure 1E, and different sessions varied in a randomly assigned resting way. Each session consisted of 400 trials in total, distributed equally in two runs. At the beginning as well as at the end of each session, subjects were required to fill the questionnaires independently. A MI task of standard left vs. right was conducted in each trial (Meng et al., 2017; Meng and He, 2019). Four hundred trials were separated half-and-half by a resting period (or immediately continued in no-rest session, which means run-2 was performed instantaneously after run-1 in no-rest session). During the rest period, a customized BCI2000 (Schalk et al., 2004) application showed a green cross at the center of the screen in the eyes-open rest condition or played an audio stimulus in the eyes-closed rest condition. The screenshots of four different stages in a single trial are shown in Figure 1D. In each MI task trial, there were four phases included: a 2-s “Pre Feedback” period after the target appeared; a 3-to-6-s “Feedback” period after cursor appeared; a 0.5-s “Post Feedback” period after the cursor hits or misses the target or a 6-s limit passed without a hit or a miss; and a 3.5-s “Inter-trial Interval” with a black screen between each trial. Subjects were required to control the cursor to hit the correct target as quickly as they can (through performing the kinesthetic motor imagination from a first-person perspective) (McFarland and Wolpaw, 2008).

Three different resting conditions were designed as follows: in the no-rest condition, 201st to 400th trials were immediately followed after the first 200 trials. The eyes-open rest condition is shown in Figure 1B, and the subjects were required to stare at the green cross on the screen and maintain a stable sitting position in a period of 16 min. The eyes-closed rest condition is shown in Figure 1C. During the 16 min of eyes-closed rest, the subjects were required to keep their eyes closed and sit still. Additionally, in order to engage the subject involved in the task during the eyes-closed rest, the subject should step on the pedal according to an audio stimulus. The sound of a “beep” stimulus was randomly played 14–23 times within 16 min (uniformly distributed), and the pedal’s input was simultaneously recorded by the BCI2000 software.

### Data Processing

#### Questionnaire

Each question in SSSQ has a score between 0 and 5, while the item in MFI-20 has a score between 0 and 20. In order to reduce the influence of individuals’ subjective differences, the questionnaire scores before each session were regarded as a baseline. The difference of the questionnaire scores between prior to and post each session was calculated to evaluate the changes of mental states.

#### Brain–Computer Interface Behavioral Performances

In the online experiments, the signals of C3 and C4 (including surrounding channels) were processed by a small Laplacian spatial filter (McFarland et al., 1997), and an autoregressive (AR) method (McFarland and Wolpaw, 2008) was used to estimate the alpha-band (8–13 Hz) power spectrum. Then, the difference of spectrum between C3 and C4 was normalized (the power value minus its average value of the past 18 s and then divided by its SD in this duration). Here, the normalizer was performed as an adaptive classifier, and it was used to counteract the adverse effect of EEG signals’ non-stationary. Finally, the normalized feature was used to control the speed of a circular cursor as feedback. There were three possible results for the control of each trial: hit, miss, or abort.

As the BCI performance criteria, percent valid correct (PVC) (Wolpaw and Wolpaw, 2012; Meng et al., 2016) and information transfer rate (ITR) (Wolpaw et al., 2000; Gao et al., 2014) were calculated. The PVC was the percent of hit trials divided by the summation of hit and miss trials. ITR was calculated based on PVC and the completion rate. Both PVC and ITR were calculated every 25 trials as a block. And ITR was converted into the average information transferred per trial, which made ITR values also between 0 and 1. Additionally, in each session, two runs’ PVC and ITR were separated equally in the middle (200 trials finished), and then the prior and post resting blocks were compared to assess the BCI performance variation within a session.

#### Electrophysiology Analysis

**FIGURE 2:**
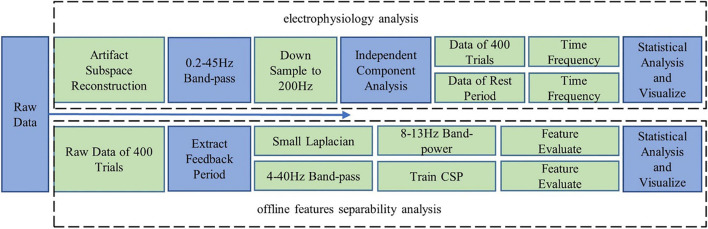
Data analysis plan: electrophysiology analysis; offline features separability analysis.

As shown in Figure 2, EEG data were analyzed with a MATLAB toolbox EEGLAB (Delorme and Makeig, 2004). First, artifacts were corrected with the Artifact Subspace Reconstruction approach (Chang et al., 2018). Second, the signals were band-pass filtered into 0.2–45 Hz, then the data were downsampled to 200 Hz, and independent component analysis [based on Tony Bell’s infomax algorithm (Bell and Sejnowski, 1995)] was performed on the data of each session to remove artifacts further.

Time–frequency analysis was performed to obtain logarithmic spectral power density. For the convenience of visualization and statistical analysis, the power density value was segmented into 1-s epochs for rest state or a 2-s epoch in each control trial (0–2 s after cursor appeared) and then averaged within a frequency band of interest to get the mean power spectrum for each epoch. Further, changes of band power were visualized in each channel and brain topography, with statistical significance thresholding (corrected *p*-value < 0.05). Similarly, the event-related desynchronization (ERD) results were also based on the time–frequency analysis, with a 2-s baseline period (2 s before the target appeared) subtracted.

The ERP was extracted from the data preprocessed by independent component analysis. Two seconds of baseline was subtracted from the control data of each trial. Then, an average of signal amplitude after the target and cursor appeared was calculated, respectively (ERP results are given in the Supplementary Material).

#### Offline Feature Separability

The online BCI performances provided us a direct indicator of the fatigue effects. However, the adaptive classifier might hide subtle variations of EEG features (the normalizer; see section “Brain–Computer Interface Behavioral Performances”). As an additional index, offline feature separability was computed to evaluate the discriminative power of the MI features. Both band power (processed by the small Laplacian spatial filter used in the online experiment) and CSP features were extracted from the raw EEG signals (channels: FC3, FC4, C5, C3, C1, C2, C4, C6, CP3, and CP4; time period: 0–2 s after cursor appeared). Then the Fisher score (Talukdar et al., 2019) and Kullback–Leibler (Shenoy et al., 2006) divergence were computed. The process is shown in Figure 2.

#### Statistical Analysis

Non-parametric hypothesis testing methods were used in this study, due to the unknown distribution of the data and a limited number of subjects (most of the results did not meet the normal distribution). The Scheirer–Ray–Hare test (Scheirer et al., 1976) was performed for the data reflecting the mental state changes. The Scheirer–Ray–Hare test is a non-parameter alternative to multifactorial repeated measures ANOVAs, and the two within-subject factors were rest condition and measured time (before or after the rest) in this study. Then, as a *post hoc* test that compares the individual groups in pairs, the sign test was used to assess the variation within each session and the variation differences between sessions (rest conditions). The BCI behavioral performances were calculated, and the performance results before rest were matched with the results after rest to perform the Scheirer–Ray–Hare test and the sign test. Additionally, the Scheirer–Ray–Hare test and the sign test were also used to assess whether the electrophysiology features significantly changed during the MI-BCI operation. Meanwhile, statistical analysis of every topographic map was corrected by the Benjamini and Hochberg method (Benjamini and Hochberg, 1995), based on the number of channels (63 channels).

## Results

### Questionnaire

**FIGURE 3:**
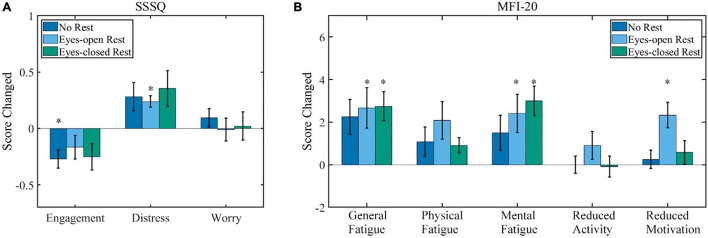
The results of the questionnaire (scores after experiment minus scores before). **(A)** The SSSQ (0–5 each state) score variations after the experiment. **(B)** The MFI-20 (0–20 each state) score variations after the experiment. Error bars indicate the standard error of mean (SEM) scores. ^∗^*p* ≤ 0.05. SSSQ, short stress state questionnaire; MFI, multidimensional fatigue inventory.

The average group difference of the SSSQ scores between the pre- and post-BCI training is shown in Figure 3A. For all of the three states in Figure 3A, the Scheirer–Ray–Hare test indicated the following: the main effects of the rest condition were not significant (*p* = 0.28, 0.53, and 0.36, respectively); the main effects of the measured time (before or after the experiment) were significant on “Engagement” and “Distress” (*p* = 0.02, 0.01, and 0.52, respectively); the interaction effects of the rest condition and measured time were not significant (*p* = 1.00, 0.97, and 0.92, respectively). Further *post hoc* test (sign test) showed detailed variations. The average scores of “Engagement” decreased in all three sessions, and the statistical analysis revealed that the difference was significant in the no-rest session. The variation of “Engagement” was −0.27 ± 0.08 [average ± standard error of mean (SEM), *p* = 0.01] in the no-rest session; −0.17 ± 0.10 (*p* = 0.11) in the eyes-open rest session; and −0.25 ± 0.12 (*p* = 0.22) in the eyes-closed rest session. The average scores of “Distress” increased in all three sessions, and there was a significant increase in the eyes-open rest session. The variation of “Distress” was 0.28 ± 0.13 (*p* = 0.18) in the no-rest session; 0.24 ± 0.05 (*p* = 0.002) in the eyes-open rest session; and 0.35 ± 0.16 (*p* = 0.11) in the eyes-closed rest session. The average score of “Worry” remains the same. The variation of “Worry” was 0.09 ± 0.08 (*p* = 0.77) in the no-rest session; −0.01 ± 0.10 (*p* = 0.75) in the eyes-open rest session; and 0.02 ± 0.12 (*p* = 0.75) in the eyes-closed rest session. Additionally, no significant difference was found between sessions (*p* ≥ 0.1) in SSSQ.

Variations of MFI-20 scores are shown in Figure 3B. For all of the five states in Figure 3B, the Scheirer–Ray–Hare test indicated the following: the main effects of the rest condition were not significant (*p* = 0.18, 0.56, 0.83, 0.53, and 0.73, respectively); the main effects of the measured time were significant on four states except “Reduced Activity” (*p* = 0.001, 0.02, 0.001, 0.80, and 0.03, respectively); and the interaction effects of the rest condition and measured time were not significant (*p* = 0.92, 0.73, 0.55, 0.79, and 0.21, respectively). A subsequent *post hoc* test (sign test) showed detailed variations. The average scores of “General Fatigue” increased in all three sessions, and there were significant variations in the eyes-open rest and eyes-closed rest. The variation of “General Fatigue” was 2.6 ± 2.5 (*p* = 0.22) in the no-rest session; 3.5 ± 3.3 (*p* = 0.02) in the eyes-open rest session; and 2.9 ± 2.2 (*p* = 0.001) in the eyes-closed rest session. “Mental Fatigue” also increased in all three sessions, and there were significant variations in the eyes-open rest and eyes-closed rest. The variation of “Mental Fatigue” was 1.4 ± 3.5 (*p* = 0.11) in the no-rest session; 2.4 ± 3.3 (*p* = 0.008) in the eyes-open rest session; and 3.0 ± 2.6 (*p* = 0.001) in the eyes-closed rest session. The state “Physical Fatigue” also increased in all the sessions but was not statistically significant (*p* ≥ 0.05). The state “Reduced Motivation” only significantly increased 1.5 ± 0.8 (*p* = 0.001) in the eyes-open rest session, but others remained almost unchanged (*p* ≥ 0.1) for both “Reduced Activity” and “Reduced Motivation.” Meanwhile, only the state “Reduced Motivation” was significantly different between the no-rest and eyes-open rest sessions (*p* = 0.02) in MFI-20; the other differences between sessions were not significant (*p* ≥ 0.1).

### Brain–Computer Interface Behavioral Performance

**FIGURE 4:**
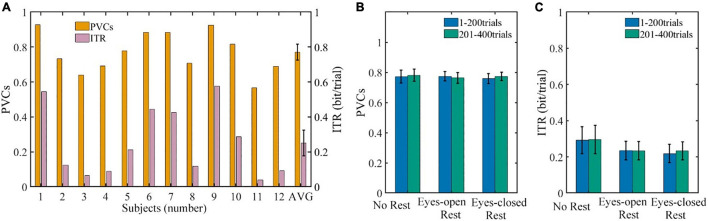
The BCI performances. **(A)** The performances of each subject (numbered) and average performance of all the subjects (AVG). **(B)** The PVCs of 1–200 trials compared with 201–400 trials for all the subjects. **(C)** The ITR per trial of 1–200 trials compared with 201–400 trials for all the subjects. Error bars indicate the SEM. BCI, brain–computer interface; PVC, percent valid correct; ITR, information transfer rate; SEM, standard error of mean.

The average BCI performances of all the 12 subjects in three sessions are shown in Figure 4A. The average PVCs of all subjects were well above chance level. The PVC ranged from 56.74% (subject 11) to 92.84% (subject 1), and the average PVC for all subjects was 77.1% ± 4.6%. The average ITR (in bit per trial) for all the subjects was 0.251 ± 0.074 bit/trial. Similar to the PVC, the ITR ranged from 0.039 (subject 11) to 0.545 bit/trial (subject 1). Additionally, a significant difference in performance between subjects was found.

The PVC and ITR of each session prior to and post to the resting period are shown in Figures 4B,C, respectively. Each session contained all of the subjects’ data and was divided into 1–200 trials and 201–400 trials according to the resting period. For the PVCs in Figure 4B, the Scheirer–Ray–Hare test indicated that the main effect of the rest conditions, the main effect of measured time, and the interaction effect were not significant (*p* = 0.52, 0.81, and 0.85, respectively). Similarly, the three effects above on ITR shown in Figure 4C were not significant as well (*p* = 0.48, 0.66, and 0.89, respectively). Furthermore, comparisons within each session (before and after rest) and between different sessions (rest conditions) were assessed with a *post hoc* sign test, respectively. Still, none of the results was significant (*p* ≥ 0.08), which means that the effects of the rest conditions and measured time were not significant on the PVC and ITR.

### Electrophysiology During the Motor Imagery Task

#### Band Power Change During the Motor Imagery Task

Band power change was found during the MI task, which supported that the experiment was long enough to induce electrophysiology changes. Meanwhile, the changes after a various duration of rest revealed different effects of rest on the band powers.

**FIGURE 5:**
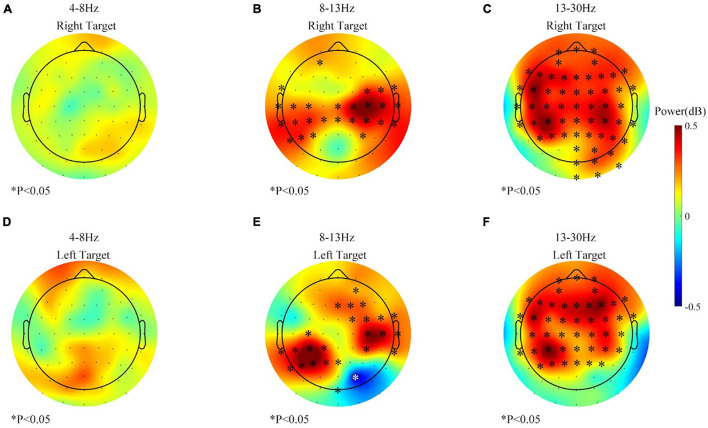
The band power changes during 1–120 trials (61–120 vs. 1–60, feedback period) of all the sessions. **(A)** Theta (4–8 Hz) power change of the right target. **(B)** Alpha (8–13 Hz) power change of the right target. **(C)** Beta (13–30 Hz) power change of the right target. **(D)** Theta (4–8 Hz) power change of the left target. **(E)** Alpha (8–13 Hz) power change of the left target. **(F)** Beta (13–30 Hz) power change of the left target. ^∗^*p* ≤ 0.05; both black and white stars were used for better visualization.

Band power change at the beginning might be different than the change across the whole session. Thus, band power changes in the feedback period of the first 200 trials from three sessions (before the rest period) were first pooled together and analyzed. And the result began to show noticeable changes after 120 trials (about 18–24 min), as shown in Figure 5. No significant difference was found in the theta-band (4–8 Hz) power. But the alpha-band (8–13 Hz) power in channels located at the sensorimotor area had significantly increased. On the contrary, the beta-band (13–30 Hz) power over the frontal area increased the most.

**FIGURE 6:**
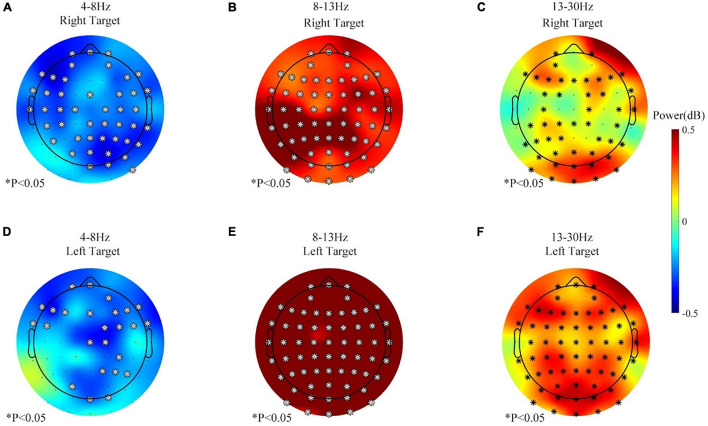
The band power changes during the no-rest session, 201–400 vs. 1–200 trials, feedback period. **(A)** Theta (4–8 Hz) power change of the right target. **(B)** Alpha (8–13 Hz) power change of the right target. **(C)** Beta (13–30 Hz) power change of the right target. **(D)** Theta (4–8 Hz) power change of the left target. **(E)** Alpha (8–13 Hz) power change of the left target. **(F)** Beta (13–30 Hz) power change of the left target. ^∗^*p* ≤ 0.05.

Second, all the 400 trials (60–80 min) were pooled together and analyzed for the no-rest session since no separate rest period was involved in this session. Figure 6 displays the change of the theta-, beta-, and alpha-band power for a long time BCI operation (400 trials, more than 60 min) in the no-rest session during the feedback period. In Figure 6, both the right and left targets caused the theta (4–8 Hz) power to decrease in some channels, while the beta (13–30 Hz) power increased instead. However, the alpha (8–13 Hz) power increased the most, statistically significant in all 63 channels after the correction (Benjamini and Hochberg method).

**FIGURE 7:**
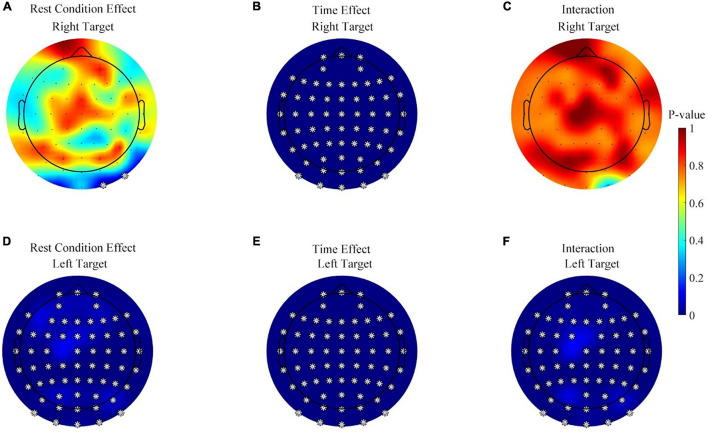
Statistical analysis results for the effects of rest conditions, the measured time, and the interaction on alpha power in the feedback period. **(A)** The effect of rest conditions during the right target. **(B)** The effect of the measured time during the right target task. **(C)** The effect of the interaction during the right target task. **(D–F)** The effect of the rest conditions, measured time, and their interaction during the left target, respectively. *p ≤ 0.05.

Third, considering that the alpha (8–13 Hz) power was used for the online control, the change of the alpha power may influence the online BCI performance. In contrast, the theta and beta powers do not directly affect the performance. Thus, it was more necessary to perform a detailed analysis of the alpha power than the theta and beta powers, to find out the effects of rest condition, time, and interaction on band power. Figure 7 displays the effects of rest conditions, the measured time, and their interaction on the alpha power during the feedback period (the first 2 s after the cursor appeared, and the alpha power analysis for baseline period was given in Supplementary Material). Data from the three sessions were used in this analysis. The color map in Figure 7 indicates the *p*-value of the Scheirer–Ray–Hare test on each channel where a corrected *p*-value below 0.05 (Benjamini and Hochberg method) was marked with a star. The effects of rest conditions were significant on the left target task over the whole brain, as shown in Figure 7D, but only two channels were significant on the right target task, as shown in Figure 7A. Meanwhile, the effect of the measured time was significant on both the right and left target tasks, as shown in Figures 7B,E.

Furthermore, Figure 7F shows that the interaction effects between rest conditions and time were significant on the left target task. All of the 63 channels popped up in the significance analysis. However, Figure 7C displays no significant interaction on any channel, during the right target task.

**FIGURE 8:**
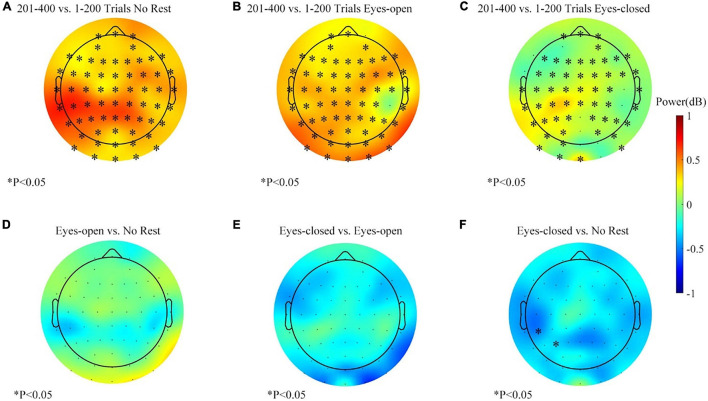
The alpha-band (8–13 Hz) power changes (201–400 vs. 1–200 trials, feedback period) in three sessions for the right target task. **(A)** Alpha power changes during the no-rest session. **(B)** Alpha power changes during the eyes-open rest session. **(C)** Alpha power changes during the eyes-closed rest session. **(D)** Differences of band power change between the eyes-open session and the no-rest session. **(E)** Differences of band power change between the eyes-closed session and the eyes-open session. **(F)** Differences of band power change between the eyes-closed session and the no-rest session. ^∗^*p* ≤ 0.05.

The Scheirer–Ray–Hare test support that the effects of rest conditions and the measured time were significant, but more detailed comparisons were necessary to investigate how the conditions of the rest/rest-conditions influence the alpha power change. The alpha-band power variations for each session in the feedback period are shown in Figures 8A–C. Data of the right target task were equally separated to contrast the variation before and after the resting period. The alpha power of most channels increased in the three sessions. However, the increase was weaker in the eyes-closed rest session. Figures 8D–F show the comparison of differences between sessions. At the parietal–occipital area, the eyes-closed resting ways had an inhibitory effect against the increasing tendency of the alpha power. Two adjacent channels popped up in the significance analysis (see Figure 8F). However, the effect of the eyes-open rest was not significant yet as compared with the no-rest session (see Figure 8D).

**FIGURE 9:**
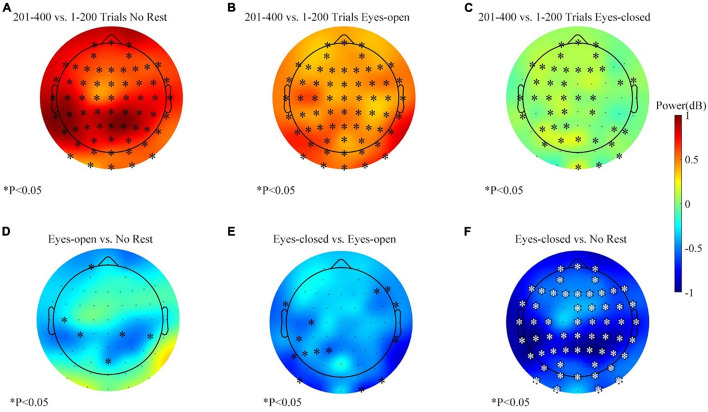
The alpha-band (8–13 Hz) power changes (201–400 vs. 1–200 trials, feedback period) in three sessions for the left target task. **(A)** Alpha power changes during the no-rest session. **(B)** Alpha power changes during the eyes-open rest session. **(C)** Alpha power changes during the eyes-closed rest session. **(D)** Differences of band power change between the eyes-open session and the no-rest session. **(E)** Differences of band power change between the eyes-closed session and the eyes-open session. **(F)** Differences of band power change between the eyes-closed session and the no-rest session. ^∗^*p* ≤ 0.05.

The alpha-band power variations during the left target task for each session are shown in Figures 9A–C. The average change of the alpha-band power was basically consistent with the right target task. However, the statistical analysis showed a subtle difference. Figures 9D–F show the differences of variation between sessions, and the results displayed more clustered channels of significance over the parietal–occipital region than those in Figures 8D–F. The effect of the eyes-closed rest was significantly different from other sessions, especially in the parietal and occipital areas (see Figure 9F). Thus, in the left target task, the eyes-closed rest had a more substantial inhibitory effect against the increase of the alpha power at the parietal and occipital areas. Meanwhile, a similar phenomenon with smaller clusters happened in the eyes-open rest session (see Figure 9D). Figure 9E displays the comparison between the eyes-closed and eyes-open sessions and proved that the effect of the eyes-closed rest was stronger.

#### Event-Related Desynchronization Results

The band power feature was based on the ERD. Thus, an equivalent analysis of ERD amplitude over two hemispheres might provide us in-depth information to understand the unchanged BCI performance.

**FIGURE 10:**
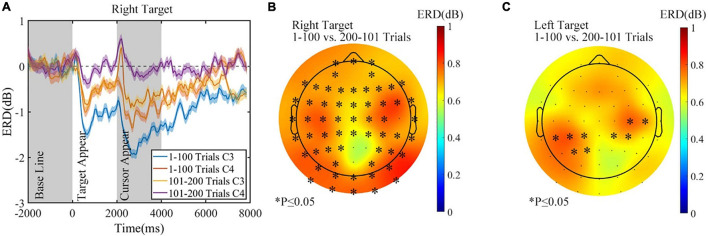
The alpha (8–13 Hz) ERD changes before rest period in all sessions. **(A)** The ERD of C3 and C4 in the first 200 trials, right target. **(B)** The ERD change before rest, right target. **(C)** The ERD change before rest, left target. ^∗^*p* ≤ 0.05. ERD, event-related desynchronization.

The experimental conditions were the same before the rest period, regardless of the assigned rest condition. Thus, data of the first 200 trials from all three sessions were pooled together. ERD of C3 and C4 in the first 200 trials for all sessions is shown in Figure 10A (right target). The absolute amplitude of ERD for both C3 and C4 was more prominent in the 1–100 trials, while C3 had a greater ERD than C4, which was reasonable during a right-hand MI task. The topographic map of variation and statistical analysis results in Figure 10B also proved that compared with that of the 1–100 trials, ERD of the 101–200 trials is more negative on both sides for the task of right target, but no significant difference was found between the two sides. Furthermore, in the control task of the left target, ERD also decreased before rest, as the average variation topographic map shown in Figure 10C, but less significant channels emerged.

#### Offline Feature Separability

**FIGURE 11:**
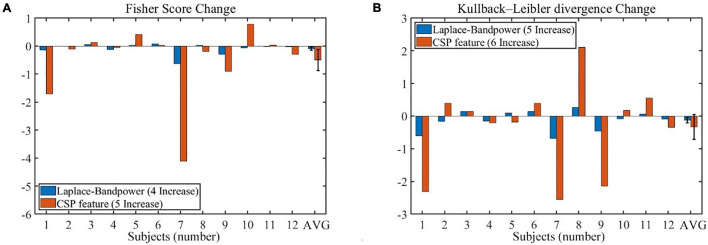
The change of offline features separability during the no-rest session (201–400 vs. 1–200 trials). **(A)** The Fisher score changes of band power feature and CSP feature. **(B)** The Kullback–Leibler divergence changes of band power feature and CSP feature. Error bars indicate the SEM. CSP, common spatial pattern; SEM, standard error of mean.

The offline feature separability could supplement the online performance evaluation and may further verify the unchanged BCI performance results. As shown in Figure 11, the change of the offline feature separability depends on subjects. The four feature separability indexes seemed to decrease in the no-rest session, but none was statistically significant. In addition, the number of increased subjects is shown in the legend of Figure 11, which had a chance level of around six increases (4/12, 5/12, or 6/12 subjects).

### Electrophysiology During Rest

**FIGURE 12:**
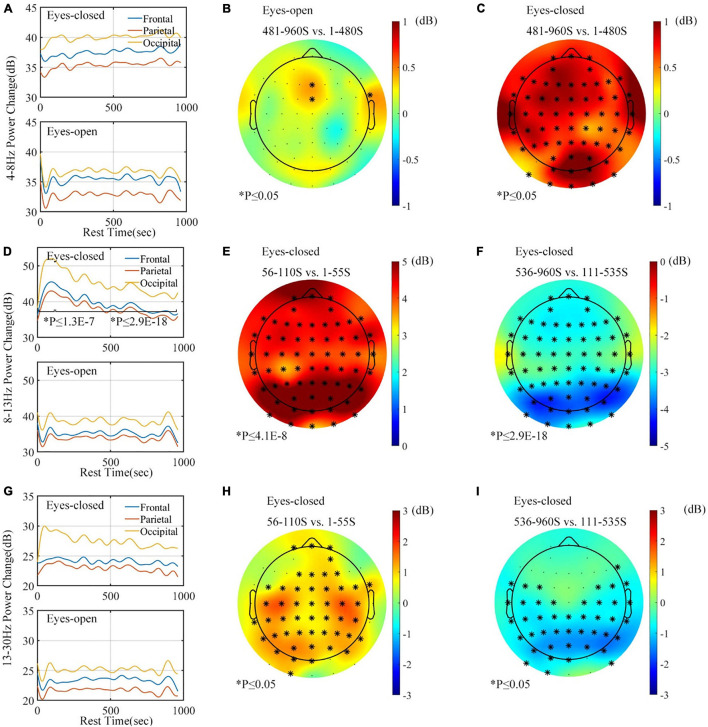
Band power analysis for rest task (frontal: FPx AFx Fx; parietal: FCx Cx CPx; occipital: Px POx Ox Ix). **(A)** Theta-band (4–8 Hz) power graph according to brain area. **(B)** Topographic map of theta (4–8 Hz) variation during eyes-open rest. **(C)** Topographic of theta (4–8 Hz) variation during eyes-closed rest. **(D)** Alpha-band (8–13 Hz) power graph according to brain area. **(E)** Topographic of alpha (8–13 Hz) variation during the first 110 s of eyes-closed rest. **(F)** Topographic of alpha (8–13 Hz) variation during the next 850 s of eyes-closed rest. **(G)** Beta-band (14–24 Hz) power graph according to brain area. **(H)** Topographic map of beta (14–24 Hz) variation during the first 110 s of eyes-closed rest. **(I)** Topographic map of beta (14–24 Hz) variation during the next 850 s of eyes-closed rest. ^∗^*p* ≤ 0.05.

All of 12 subjects correctly responded to the audio stimulus during the eyes-closed rest, by stepping on the pedal within 3 s after the “beep” sound. It meant that none of the subjects fell asleep during the rest period. An interesting phenomenon was found during the rest period. The theta-band (4–8 Hz) power significantly increased during the eyes-closed rest, but the band power remained basically unchanged during the eyes-open rest. Figure 12A shows that the plot of the theta power changes in different brain areas under different resting ways (Figure 12 includes data of 11 subjects because the data of a subject during eyes-open rest contain artifacts). Figures 12B,C show the average variations of the theta-band power between the early and late stages of the rest period under different rest conditions. The theta-band power in all channels increased in the eyes-closed rest condition, while that power in the eyes-open rest condition remained unchanged in most channels.

As Figure 12D shows, although the alpha power under the eyes-open rest demonstrated no significant change across the rest period, the change of the alpha-band power displayed a different tendency under the eyes-closed rest. During the eyes-closed rest, an increasing tendency was found in the first 110 s, while the last 850 s had a decreasing tendency. This phenomenon showed statistical significance in all channels; Figures 12E,F also show that the variation was stronger in the occipital area. Note that both Figures 12E,F show the variation of the eyes-closed rest.

The beta-band power change is presented in Figures 12G–I. Band power remained unchanged in the eyes-open rest condition, while the eyes-closed rest caused an increase and subsequent decrease only in the parietal–occipital area. This variation was similar to the alpha-band power, but the amplitude of increase and decrease was smaller.

In summary, we found that general fatigue, mental fatigue, and distress have significantly increased while engagement has decreased after a session of intensive MI-BCI operation (time effect). BCI performances (PVC and ITR) and offline feature separability were stable within a session. Further electrophysiological analysis revealed that the alpha and beta powers significantly increased within the session, while the theta power had slightly decreased due to repeated task trials. Furthermore, the alpha ERD amplitude decreased within a session. Additionally, the change of the alpha power was found during both the rest period and task period after the rest, but the effect of rest was not significant on altering mental states such as alleviating fatigue. Due to BCI performance, prior- and post-BCI training was not significantly changed (see the results of section “Brain–Computer Interface Behavioral Performances”), it was expected that the rest would not have effects on changing BCI performance.

## Discussion

Previous studies showed that electrophysiological signals like spectral powers are correlated with BCI performance (Blankertz et al., 2010). Both the alpha and beta bands were commonly used for MI-BCI control. However, previous studies also showed that fatigue affects both the alpha-band (Cao et al., 2014; Seo et al., 2019; Jacquet et al., 2021) and beta-band (Tanaka and Watanabe, 2011) activities. Thus, the effects of fatigue on MI-BCI are worth investigating. The change of BCI performances and electrophysiological indicators during prolonged MI-BCI operation and the rest are discussed below.

### Effects of Prolonged Motor-Imagery-Brain-Computer Interface Operation

The questionnaire results confirm the assumption that the mental state changes (especially the general fatigue and mental fatigue increased) were induced, as Figure 3 shows. The changes indicate that our prolonged MI-BCI operation was long enough to alter the mental states of users. But the results in Figure 4 show that there was no significant change in BCI performance of the cursor control task. Further analysis in Figures 5–10 revealed that the prolonged MI-BCI operation could significantly alter electrophysiological indicators. Especially, the alpha-band power increase over the whole head was consistent with the literature (Cao et al., 2014; Seo et al., 2019; Jacquet et al., 2021), which might be associated with an increased mental effort to maintain a state of alert wakefulness (Klimesch, 1999).

Furthermore, since the online control signals were extracted from the channels around C3 and C4, and based on the alpha rhythm, the modulation of the alpha rhythm may increase due to the alpha-band power increase. But the ERD results in Figure 10 demonstrated a decreasing tendency, which contradicts this speculation. Nevertheless, the online performance and offline feature separability results (see Figures 4, 11) support the notion that the increased power amplitudes and altered mental states did not significantly impact the BCI behavioral performance during the MI-BCI operation.

There might be several reasons to explain these results. First, although the previous study suggested that the strength of the SMR power is related to the MI-BCI performance (Blankertz et al., 2010) in a group of 80 subjects, the predictor of SMR was calculated from a 2-min recording under “relax with eyes open.” This predictor, obtained in a short period, neglected the dynamic property of the alpha power for an individual in a single session. In another study, a positive correlation of the beta-band power and BCI performance was found by Foong (Foong et al., 2019) in 11 subjects’ stroke rehabilitation study. In this 6-week long-term rehabilitation period, 18 sessions of BCI experiments were performed on different days per subject. However, the positive correlation between the beta-band power changes and the BCI performance may not hold in a single session. The current study showed that the power in different frequency bands calculated in a single session did not affect the performance of BCI. Thus, the previous correlative results of the alpha-band power in a brief period and the beta-band power in a long-term period did not contradict this study’s results.

Second, Talukdar et al. (2019) reported that the alpha power increases and the offline MI feature separability decreases with mental fatigue during prolonged MI tasks. In their study, no instantaneous feedback was provided during the 3 s of MI period. But in our experiment, instantaneous online feedback was provided on the screen, and an adaptive classifier (the normalizer) was implemented. Thus, subjects could adapt to the variation of BCI performance if fatigue might deteriorate the performance. Further analyses of offline feature separability did not show any significant changes, which coincided with the no change of BCI performance. However, the current analysis could not entirely exclude the complex effect of an adaptive classifier on the feature’s distribution. Thus, we have to interpret no significant change of feature separability with caution.

Meanwhile, our results demonstrated that the beta-band (13–30 Hz) power also increases during prolonged MI-BCI operation. The increase of the beta-band power was consistent with experiment 2 of Cao’s work (Cao et al., 2014) but differed from another study about the mental fatigue induced by mental arithmetic tasks (Trejo et al., 2015). Moreover, the contradictive results of the beta-band power were consistently reported in the literature (Tran et al., 2020). Therefore, it might mean that the beta power change with the fatigue might depend on how fatigue was induced.

The theta-band (4–8 Hz) power had no significant change in the first 120 trials and slightly decreased in the no-rest session across 400 trials of MI tasks. In Cao’s study, the theta power increased (in experiment 1, 2.5 min) or remained unchanged (in experiment 2, 7.2 min) as the task progresses (Cao et al., 2014); the variation of theta power change depended on the experimental design. Note that the BCI operation of Cao’s work was conducted within 10 min, which was much shorter than our study (more than 60 min), and the theta power only increased in experiment 1 with a shorter time. Meanwhile, in another study about MI-induced fatigue (non-BCI), the theta power decreased with time in 53-min knee MI experiments (Jacquet et al., 2021), which is consist with our results in Figures 6A,D (across the 400 trials, 65 min on average). Thus, the different experiment durations and BCI tasks might cause the various theta power changes.

Although we perform a separate training session before the formal data collection, the training effect might still exist. However, the randomized complete block design, which restricted the nuisance factor of training effect on the BCI performance, could potentially compare treatment impacts on MI-BCI performance as homogenous as possible. Thus, the training factor might not be a confounding factor to the current study’s results.

### Effects of Rest

During the rest period, no electrophysiological indicator change had been found during eyes-open rest; on the contrary, an alpha-band power increase and subsequent decrease appeared in the eyes-closed rest condition. These phenomena demonstrated that the rest conditions with eyes open or closed might cause different physiological activities, even though subjects kept awake during both rest conditions. Furthermore, Boytsova and Danko showed that the alpha-band power was higher in the resting state with eyes closed than eyes open (Boytsova and Danko, 2010), which was consistent with our data. But the authors mentioned neither the duration of the rest nor the increasing and decreasing processes. The long rest durations of 16 min in our study might explain the inconsistency between research results.

Interestingly, the rest process influenced the electrophysiological indicators during the rest and affected different band powers after the rest. As Figures 7–9 show, process of the rest/rest-process caused the alpha-band power to increase differently between each session. Especially at the parietal and occipital areas, both resting ways had an inhibitory effect against the increasing tendency of the alpha-band power. And this effect under the eyes-closed rest was more substantial than that under the eyes-open rest. Therefore, if the fatigue effects induced the alpha-band power increase, the inhibitory effect against the increasing tendency of the alpha-band power might reveal the positive impact of the rest against the fatigue. However, the influence of rest condition on BCI performance and mental states were not found (see sections “Questionnaire” and “Brain–Computer Interface Behavioral Performance”).

### Limitations and Future Work

It has to be acknowledged that the experimental setup in this study was only a typical demonstration for MI-BCI operation. The prolonged BCI operation includes MI, watching a screen, and feedback process. Fatigue could be induced by a combination of these factors or their interaction. We cannot precisely separate which factors caused the fatigue, which was not the focus of this study. We have utilized both the SSSQ (for assessment of task-induced subjective feelings) and the MFI-20 (for assessment of possible long-term subjective feelings) to assess the subjective fatigue feelings with the aim to provide comprehensive insights about the time-on-task influence. However, the MFI scores may lack the sensitivity to assess the subjective feelings of mental fatigue. Task-Induced Fatigue Scale (TIFS: Desmond and Matthews, 1998) or a post-task workload measure such as the NASA-TLX (Hart and Staveland, 1988) might be better in the subjective assessment of mental fatigue, which will be considered in our future work.

Further, our experiment lasts for more than 1 h in each session, but there is a 3.5-s inter-trial interval between two trials. A break of idle state is typical in an MI-BCI setup, but it might reduce the mental workload. It is worth investigating whether the results of basic cursor control tasks still hold for more complicated and challenging situations such as continuous cursor tracking (Edelman et al., 2019). Furthermore, this study was conducted in a group of healthy subjects with an average age of 22. Therefore, the current results might not easily extrapolate to the senior people and patient group.

## Conclusion

We investigated if intensive and prolonged MI-BCI operation would affect MI-BCI performance, electrophysiological indicators, and mental states in a group of 12 healthy subjects. Our results indicated that prolonged MI-BCI operation might significantly affect the electrophysiological indicators and mental states but might not affect the BCI performance and feature separability. The alpha and beta bands’ power increased across the task progression, but the alpha ERD modulation level seems to decrease. Furthermore, the eyes-closed rest caused the alpha-band power increase and subsequent decrease during a separate rest block and has a more substantial effect than the eyes-open rest on inhibiting the increase in the alpha-band power in the parietal and occipital areas. Altogether, the results of this study revealed that the fatigue effects during prolonged MI-BCI operation might not be a crucial factor for the online BCI performance even though subjective fatigue was successfully induced, and different rest conditions altered the electrophysiological indicators differently. In a typical BCI experiment of 1-h duration or a little above (two tasks of MI, with feedback), a short inter-trial interval might be enough to counteract the effect of fatigue. Therefore, an extra-long break might not be necessary for a BCI use within an hour. The present results explored the fatigue effects on MI-BCI performance and may give valuable inspiration to designing a robust MI-BCI.

## Data Availability Statement

The raw data supporting the conclusions of this article will be made available by the authors under a material transfer agreement with Shanghai Jiao Tong University, without undue reservation.

## Ethics Statement

The studies involving human participants were reviewed and approved by The Institutional Review Board of Shanghai Jiao Tong University. The patients/participants provided their written informed consent to participate in this study.

## Author Contributions

SL and JD wrote the first draft of the manuscript. JM, YS, and XZ conceived and designed the experimental paradigm. JM, SL, and JD performed the research and analyzed data. SL, JD, JM, XS, and XZ wrote the manuscript. All authors edited the manuscript.

## Conflict of Interest

The authors declare that the research was conducted in the absence of any commercial or financial relationships that could be construed as a potential conflict of interest.

## Publisher’s Note

All claims expressed in this article are solely those of the authors and do not necessarily represent those of their affiliated organizations, or those of the publisher, the editors and the reviewers. Any product that may be evaluated in this article, or claim that may be made by its manufacturer, is not guaranteed or endorsed by the publisher.
