# Supplementary material for: Exploring Fatigue Effects on Performance Variation of Intensive Brain–Computer Interface Practice
Source: Front Neurosci. 2021 Dec 2;15:773790. doi: 10.3389/fnins.2021.773790 (PMC8678598; doi:10.3389/fnins.2021.773790)
Supplement: Supplementary file 1 [file Data_Sheet_1.docx]

# Supplementary Materials

# Event Related Potential Results


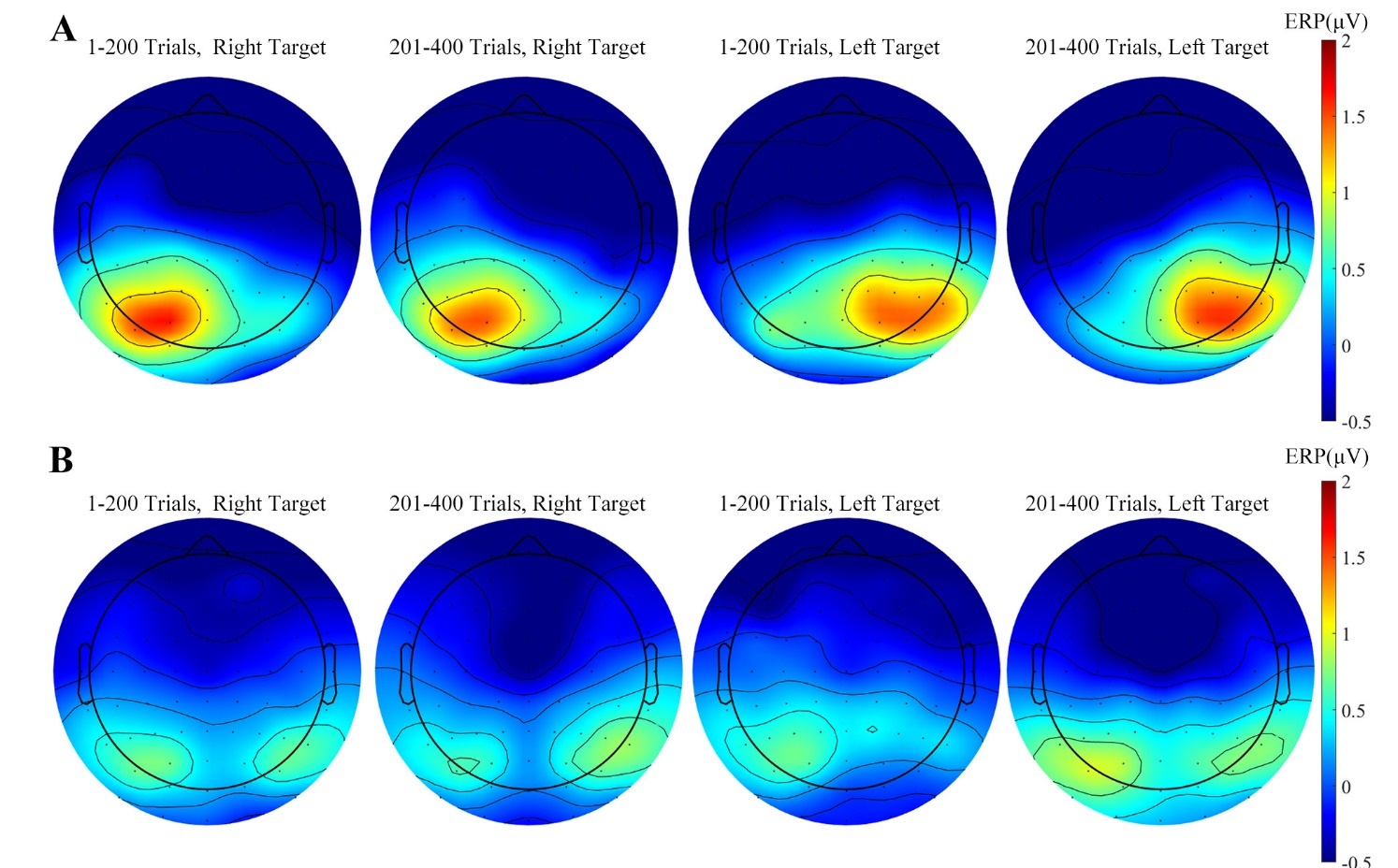


**Figure S1.** Topographic maps of average ERP amplitude. **(A)** Average ERP amplitude of P300 after Target Appeared, **(B)** Average ERP amplitude of P300 after Cursor Appeared.

The average ERP amplitude of P300 after the target appeared was given in figure [S1A,](#_bookmark30) while the average ERP amplitude after the cursor appeared was shown in figure [S1B.](#_bookmark30) The topographic map in figure [S1](#_bookmark30) was based on the data of no rest session. The ERP amplitude was mainly maximized in the occipital area, and it showed lateralization in most conditions. A right target on the screen appeared in the right visual hemifield. Hence, the maximum amplitude of the ERP appeared in the left hemisphere. The opposite relation held for the left target. There was similar but less consistent lateralization after the cursor appeared. This could be explained that as most subjects moved their sight to the target after the target appeared, and a cursor at the center of the screen appeared on the opposite side of the visual hemifield. Therefore, the lateralization still held.

Meanwhile, the average ERP amplitude for 201-400 trials was weaker than that of 1-200 trials. In order to investigate the change of ERP amplitude, statistical analysis results for ERP amplitude were shown in figure [S2.](#_bookmark31)  Averaged ERP waves of channel PO3 and PO4 in figures [S2A-B](#_bookmark31) were based on the data of no rest session since the fatigue effect was most substantial according to the previous analysis. Shadow area indicated the P300 after the target or cursor appeared. Figure [S2C](#_bookmark31) showed the average amplitude in the shadow area for right target trials in different sessions, with the error bar presenting the SEM of trials. And the left target average amplitude was shown in figure [S2D](#_bookmark31). The ERP caused by the left target cursor appears significantly increased in no rest session, but all the other statistical analyses are not significant.


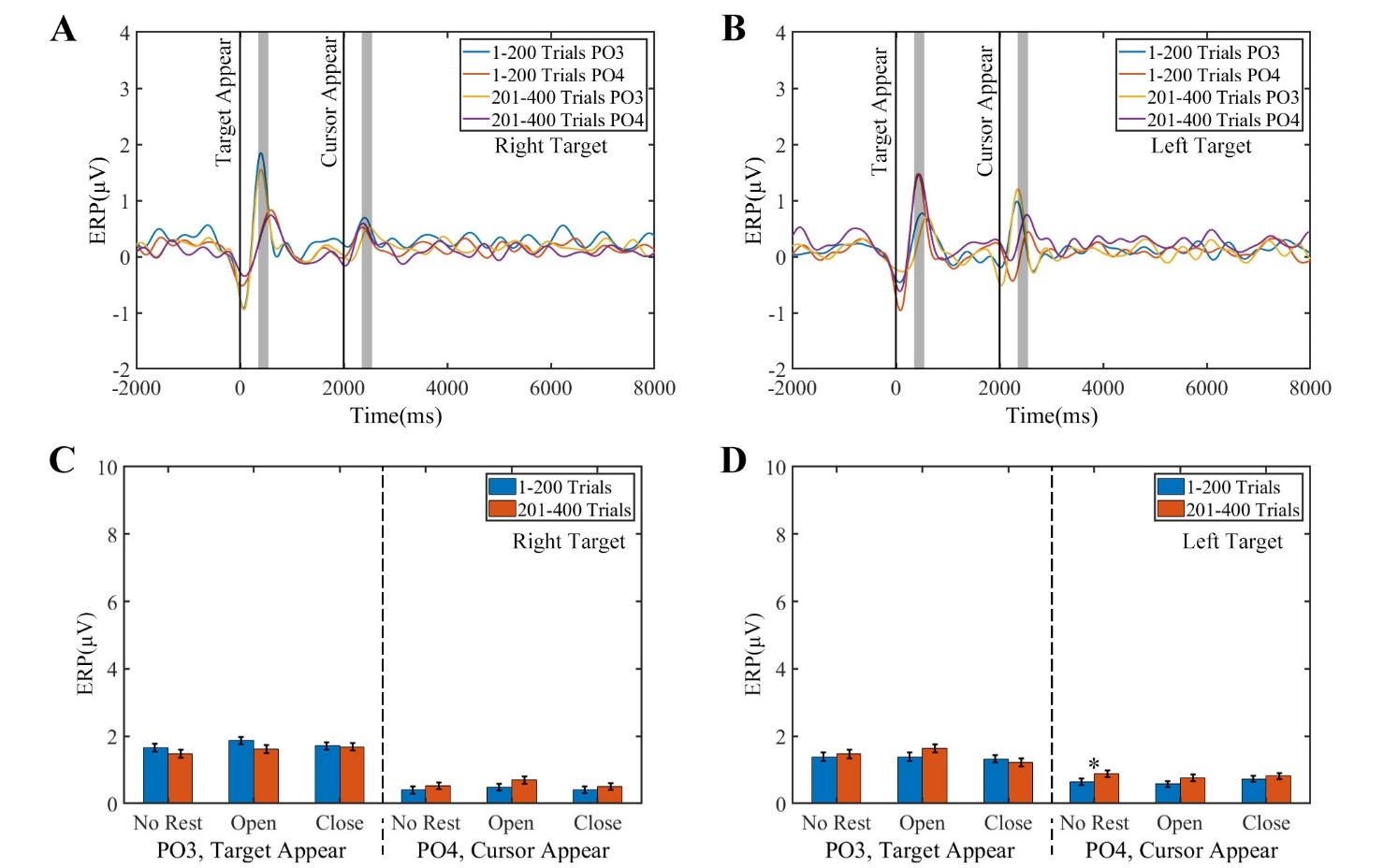


**Figure S2.** Statistical analysis results for ERP amplitude. **(A)** Average ERP wave plot for PO3 and PO4 in Right Target (based on no rest session, separated into 200 trials), **(B)** Average ERP wave plot for PO3 and PO4 in Left Target, shadow area indicated P300. **(C)** ERP amplitude for 1-200 trials and 201-400 trials for Right Target. **(D)** ERP amplitude for 1-200 trials and 201-400 trials for Left Target. (∗P < 0.05)

# Theta and Beta Power, 301-400 vs. 1-100 trials

Figure S3 displayed the change of theta and beta power for 301-400 vs. 1-100 trials in the no rest session during the feedback period, respectively. The first and last 100 trials comparison showed a subtle difference from figure 6. In figure S3, the theta (4-8Hz) power decreased in fewer channels, while the beta (13-30Hz) power increased similarly to figure 6.


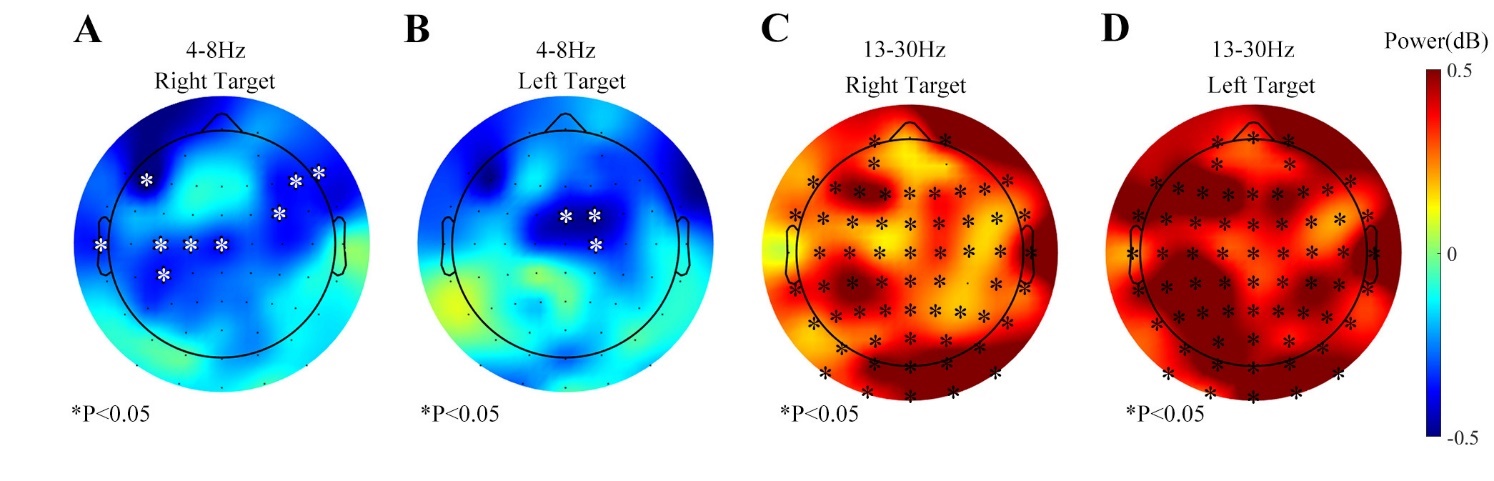


**Figure S3.** The band power changes during the no rest session, 301-400 vs. 1-100 trials, **(A)** Theta (4-8Hz) power change (right target), **(B)** Theta (4-8Hz) power change (left target), **(C)** Beta (13-30Hz) power change (right target), **(D)** Beta (13-30Hz) power change (left target). (∗P ≤ 0.05)

# Alpha power analysis for baseline periods

The effects of rest conditions, the measured time, and their interaction on the alpha power during the baseline period (the last two seconds before the target appeared) were shown in figure S4. Data from the three sessions were used in this analysis. The color map in figure S4 indicated the P-value of the Scheirer–Ray–Hare test on each channel where a corrected P-value below 0.05 (Benjamini and Hochberg method) was marked with a star. Compared to the corresponding results in the feedback period in figure 7, the results of the baseline period showed more substantial effects in the right-hand target while fewer effects in the left-hand target.


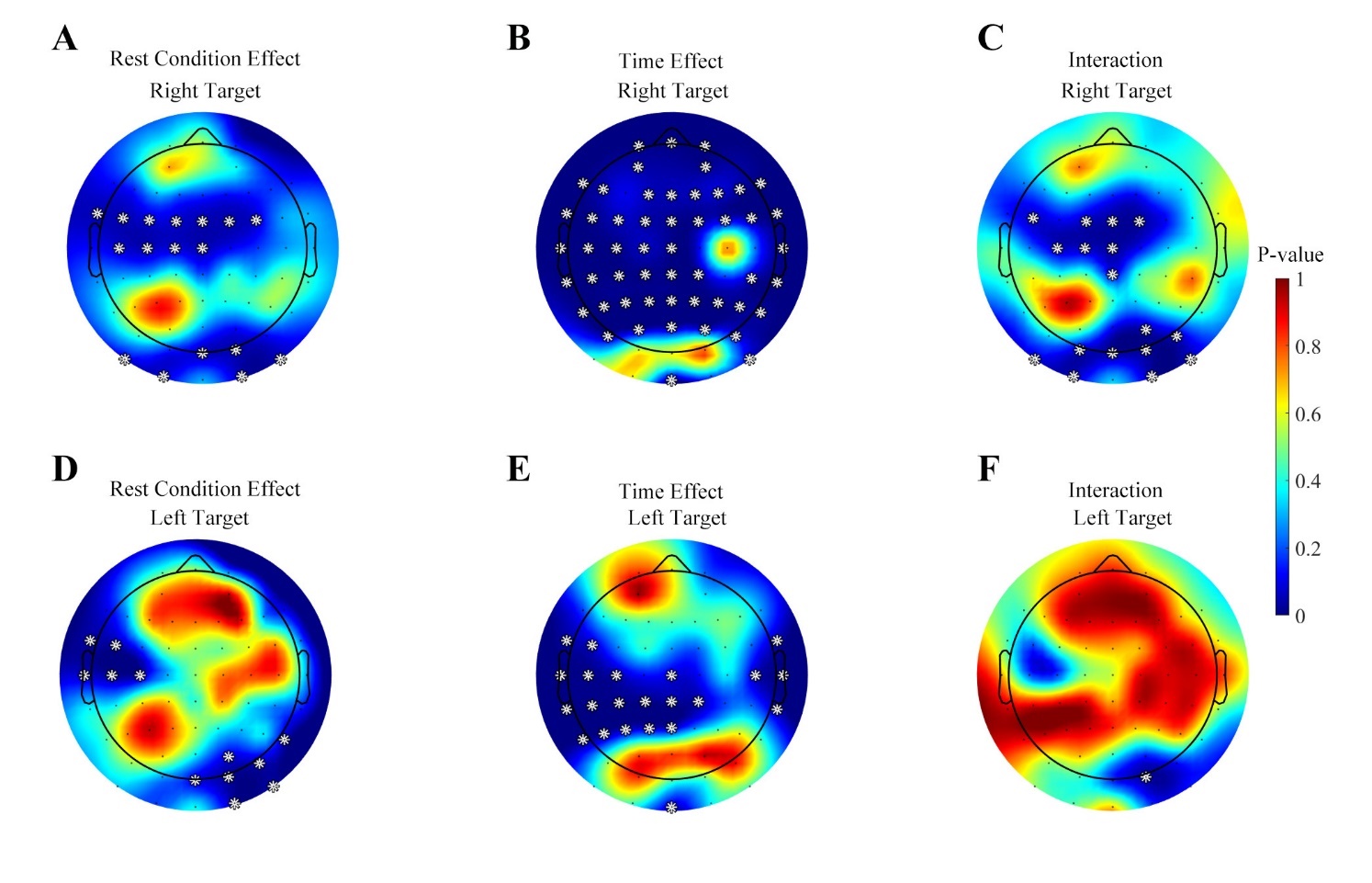


**Figure S4.** Statistical analysis results for the effects of rest conditions, the measured time, and the interaction on alpha power in the baseline period. **(A)** Effect of rest conditions during the right target, **(B)** Effect of the measured time during the right target task, **(C)** Effect of the interaction during the right target task, **(D-F)** Effect of the above factors during the left target, respectively. (∗P ≤ 0.05)


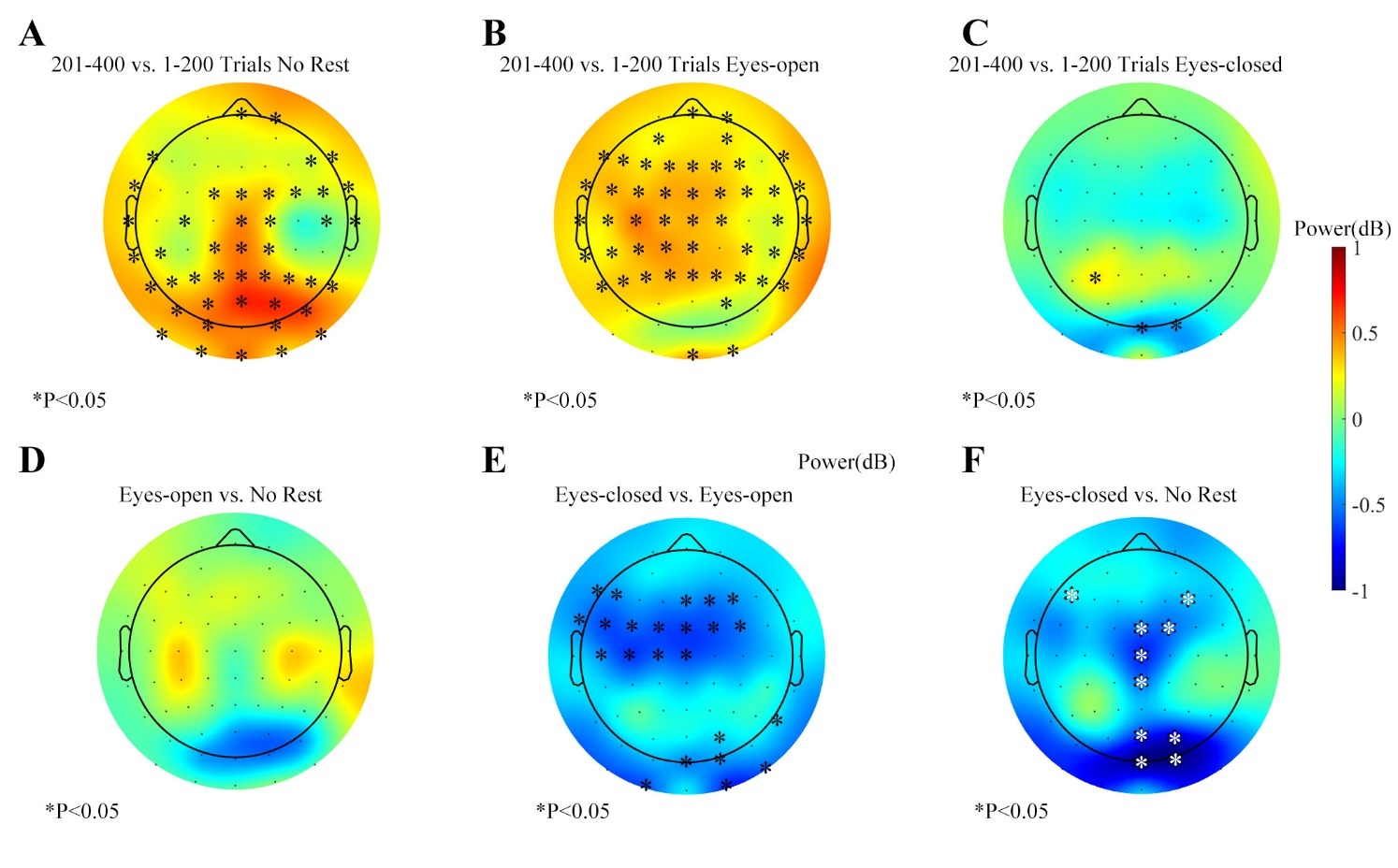


**Figure S5.** The alpha-band (8-13Hz) power changes (201-400 vs. 1-200 Trials, baseline period) in three sessions for the right target task. **(A)** Alpha power changes during the no rest session, **(B)** Alpha power changes during the eyes-open rest session, **(C)** Alpha power changes during the eyes-closed rest session, **(D)** Differences of band power change between the eyes-open session and the no rest session, **(E)** Differences of band power change between the eyes-closed session and the eyes-open session, **(F)** Differences of band power change between the eyes-closed session and the no rest session.

The alpha power changes during the baseline period were shown in figures S5 and S6 for the right and left targets. Compared to the results of the feedback period in figures 8 and 9, changes during the baseline period were similar, and the alpha power also increased the most in the no rest session. But the comparison of differences between sessions showed a more substantial inhibiting effect at the occipital lobe in the eyes-closed rest session.


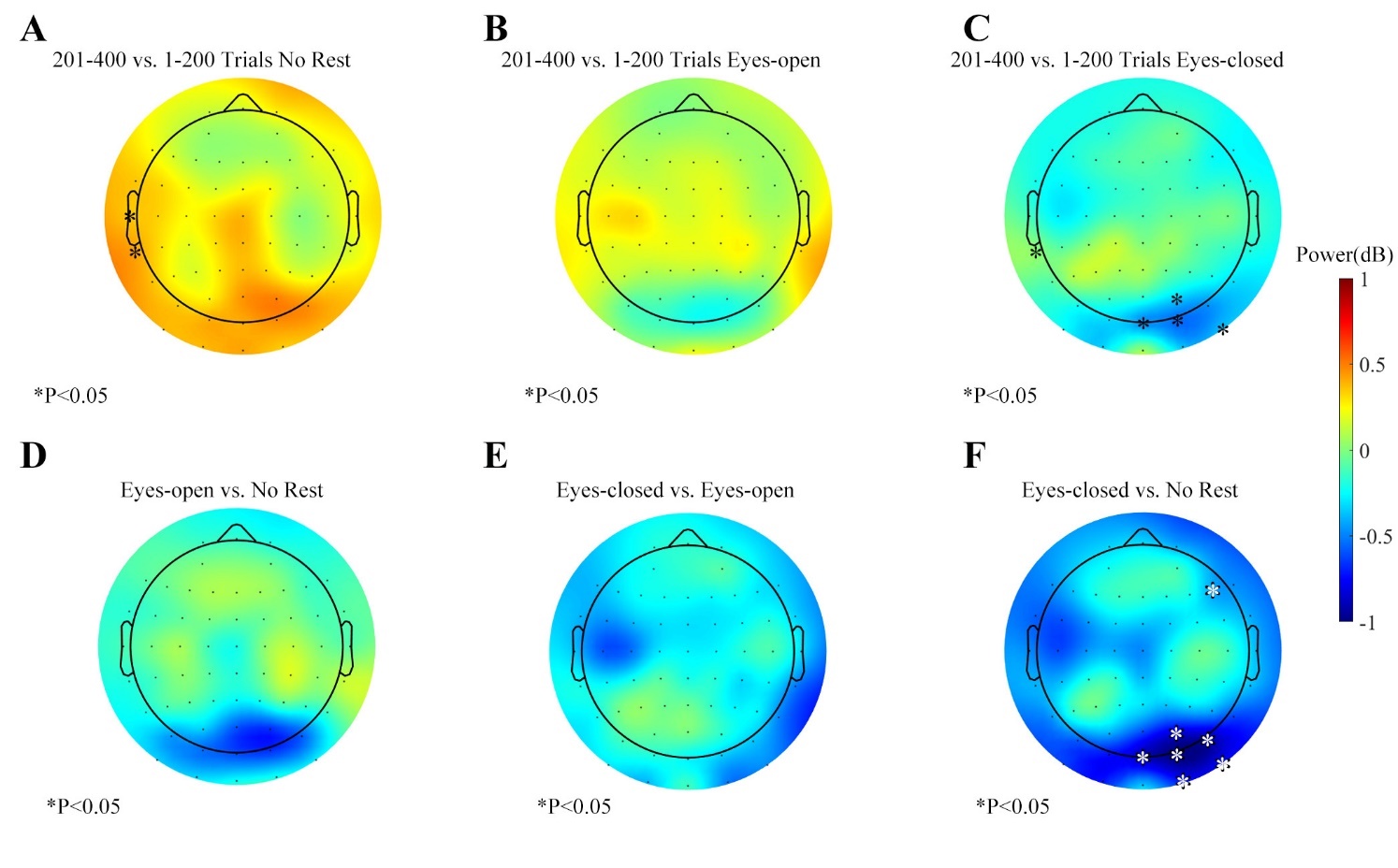


**Figure S6.** The alpha-band (8-13Hz) power changes (201-400 vs. 1-200 Trials, baseline period) in three sessions for the left target task. **(A)** Alpha power changes during the no rest session, **(B)** Alpha power changes during the eyes-open rest session, **(C)** Alpha power changes during the eyes-closed rest session, **(D)** Differences of band power change between the eyes-open session and the no rest session, **(E)** Differences of band power change between the eyes-closed session and the eyes-open session, **(F)** Differences of band power change between the eyes-closed session and the no rest session.

The alpha ERD absolute amplitude changes were shown in figures S7 and S8, in a range of 400 trials (1-200 vs. 201-400 Trials) for the right and left target, respectively. Compared with figure 10, figures S7 and S8 were based on three sessions separately. With fewer trials in each session, the statistical analysis results showed less significance. The alpha ERD was weaker in 201-400 trials, consistent with a shorter duration of the results (figure 10). Furthermore, both the contrast result between eyes-open rest and No rest and the contrast result between eyes-closed rest and No rest showed negative values. It suggested that both the eyes-open rest and eyes-closed rest appeared to inhibit the ERD absolute amplitude decrease at the parietal-occipital area. However, the statistical analysis showed that the effects of rest conditions on ERD amplitude were not significant.


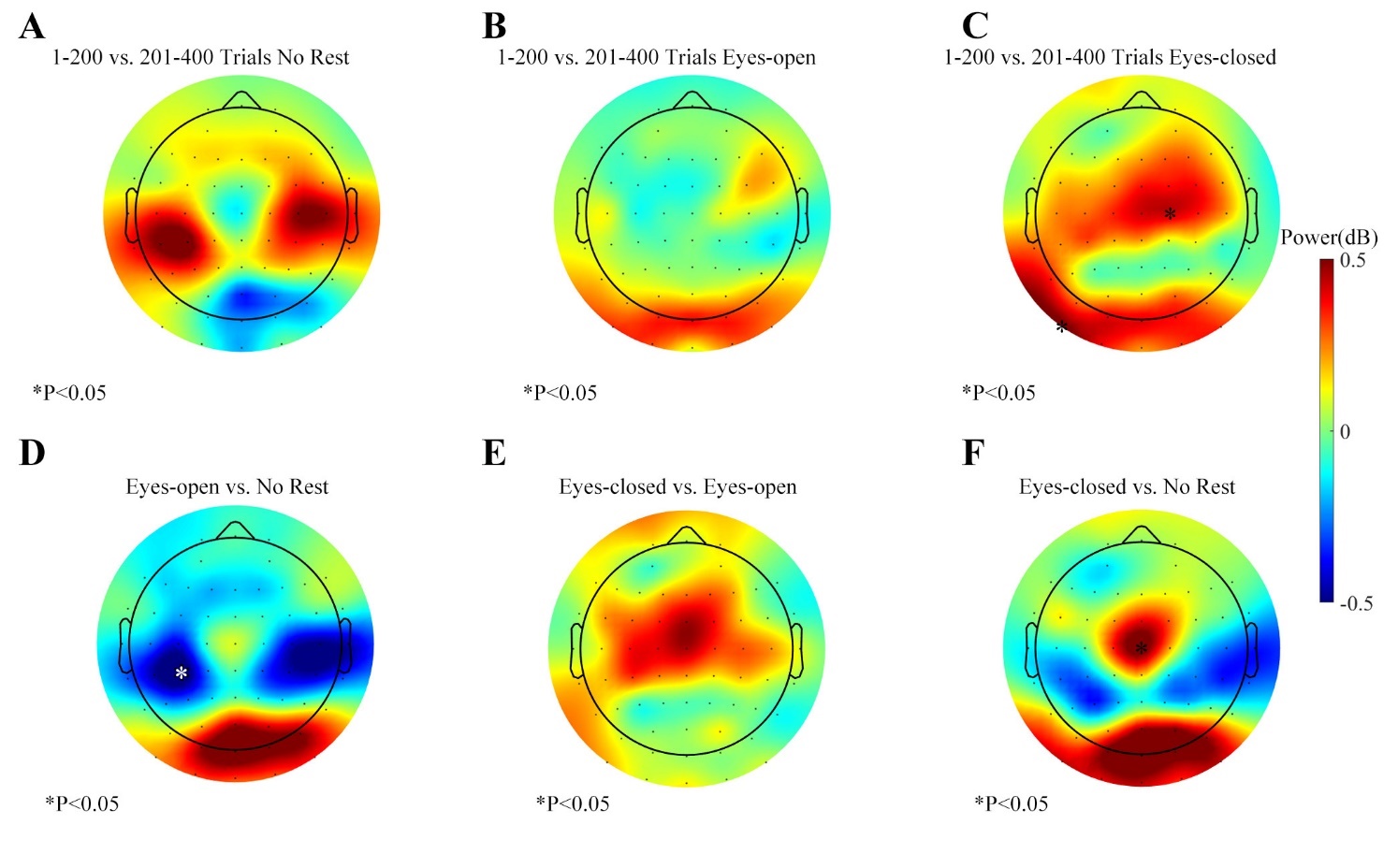


**Figure S7.** The alpha-band (8-13Hz) ERD changes (1-200 vs. 201-400 Trials) in three sessions for the right target task. **(A)** ERD changes during the no rest session, **(B)** ERD changes during the eyes-open rest session, **(C)** ERD changes during the eyes-closed rest session, **(D)** Differences of ERD change between the eyes-open session and the no rest session, **(E)** Differences of ERD change between the eyes-closed session and the eyes-open session, **(F)** Differences of ERD change between the eyes-closed session and the no rest session.


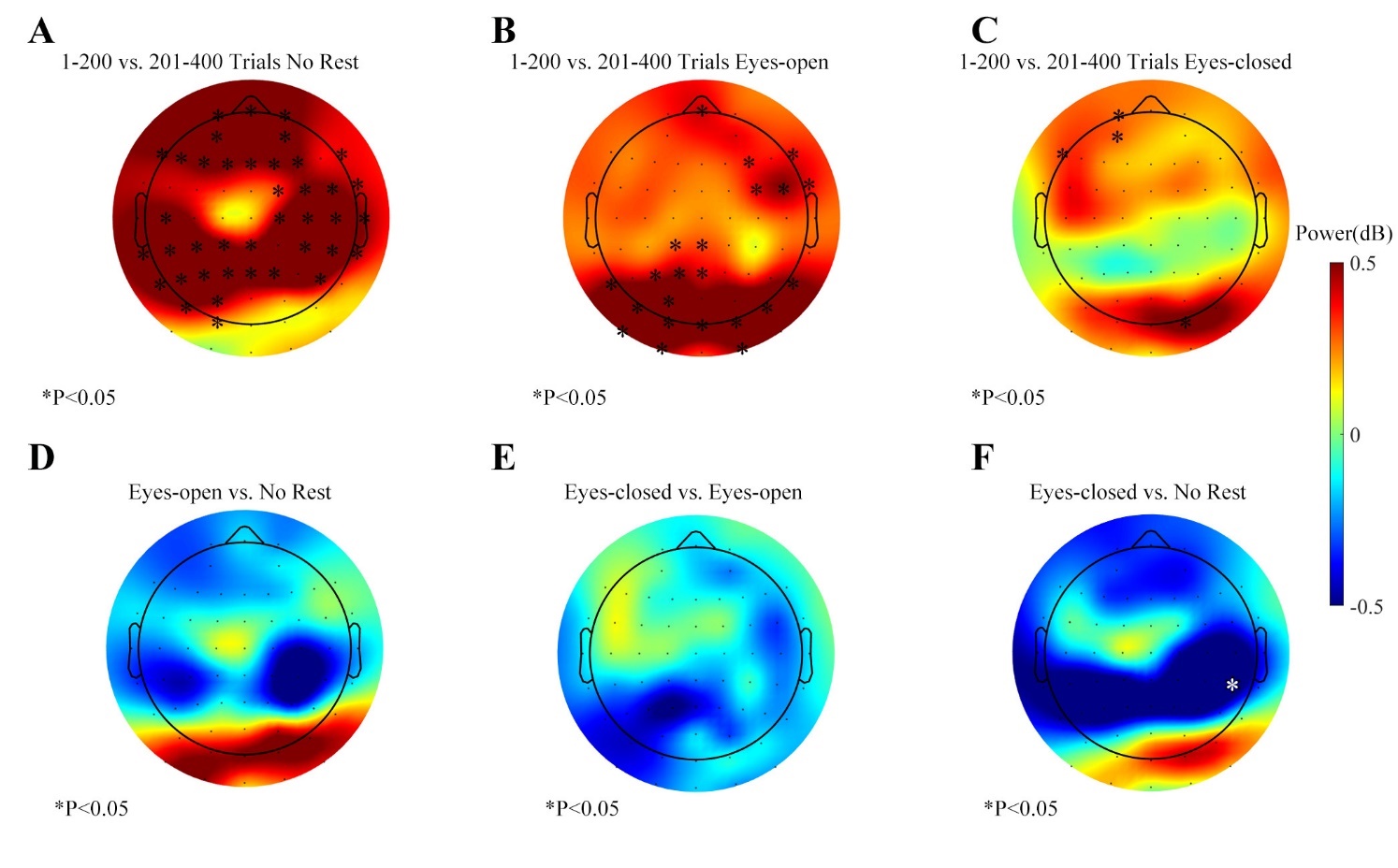


**Figure S8.** The alpha-band (8-13Hz) ERD changes (1-200 vs. 201-400 Trials) in three sessions for the left target task. **(A)** ERD changes during the no rest session, **(B)** ERD changes during the eyes-open rest session, **(C)** ERD changes during the eyes-closed rest session, **(D)** Differences of ERD change between the eyes-open session and the no rest session, **(E)** Differences of ERD change between the eyes-closed session and the eyes-open session, **(F)** Differences of ERD change between the eyes-closed session and the no rest session.

# CSP channel weight

Figures S9 and S10 displayed the CSP weights on the channels used in the offline feature separability analysis. The most significant CSP filters, corresponding to the first and last ordered eigenvalues, were shown in figures S9 and S10, respectively. Nevertheless, the CSP weights were the average value of 12 subjects, which indicated a common weight distribution among subjects. Thus, the results showed consistency with the prior expectation, in which C3 and C4 played the most critical roles in discriminating the left and right target tasks.

**
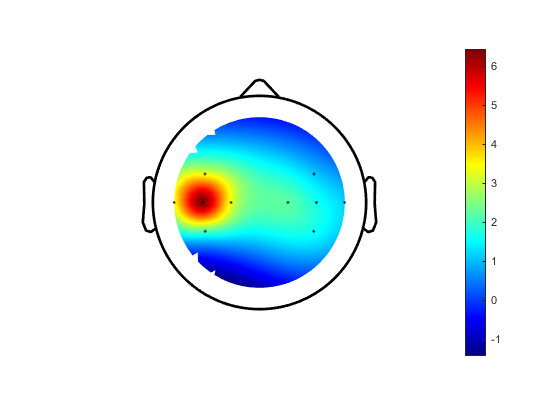
**

**Figure S9.** The CSP channel weight group 1 (first).

**
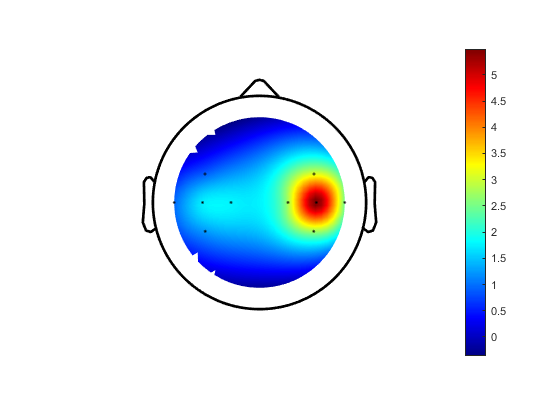
**

**Figure S10.** The CSP channel weight group 10 (last).

Table I PVC (%)

| Subject(num) | No rest | | Eyes-open rest | | Eyes-closed rest | |
| --- | --- | --- | --- | --- | --- | --- |
|  | 1-200 | 201-400 | 1-200 | 201-400 | 1-200 | 201-400 |
| 1 | 92.9 | 90.8 | 95.4 | 95.3 | 86.6 | 96.0 |
| 2 | 64.9 | 74.6 | 75.0 | 70.9 | 83.9 | 70.1 |
| 3 | 62.8 | 63.6 | 55.1 | 65.6 | 68.5 | 68.8 |
| 4 | 81.0 | 70.4 | 62.2 | 69.2 | 62.5 | 69.6 |
| 5 | 74.9 | 83.4 | 75.7 | 75.2 | 83.2 | 75.3 |
| 6 | 89.9 | 87.7 | 88.7 | 90.9 | 87.7 | 86.0 |
| 7 | 95.9 | 87.2 | 83.6 | 87.6 | 94.8 | 81.3 |
| 8 | 75.4 | 65.1 | 71.6 | 73.0 | 62.2 | 76.9 |
| 9 | 97.2 | 93.0 | 87.3 | 99.5 | 88.1 | 89.7 |
| 10 | 82.0 | 79.9 | 83.0 | 92.0 | 70.6 | 82.9 |
| 11 | 50.4 | 60.6 | 60.5 | 50.1 | 53.3 | 65.6 |
| 12 | 59.1 | 73.1 | 72.7 | 68.1 | 75.1 | 65.7 |

Table II ITR (bit/trial)

| Subject(num) | No rest | | Eyes-open rest | | Eyes-closed rest | |
| --- | --- | --- | --- | --- | --- | --- |
|  | 1-200 | 201-400 | 1-200 | 201-400 | 1-200 | 201-400 |
| 1 | 0.498 | 0.444 | 0.616 | 0.678 | 0.368 | 0.668 |
| 2 | 0.094 | 0.108 | 0.133 | 0.084 | 0.232 | 0.097 |
| 3 | 0.051 | 0.069 | 0.035 | 0.055 | 0.077 | 0.098 |
| 4 | 0.212 | 0.081 | 0.030 | 0.070 | 0.071 | 0.071 |
| 5 | 0.165 | 0.343 | 0.155 | 0.167 | 0.305 | 0.127 |
| 6 | 0.581 | 0.430 | 0.441 | 0.485 | 0.375 | 0.350 |
| 7 | 0.622 | 0.356 | 0.312 | 0.354 | 0.618 | 0.289 |
| 8 | 0.142 | 0.052 | 0.112 | 0.176 | 0.042 | 0.184 |
| 9 | 0.810 | 0.580 | 0.340 | 0.895 | 0.421 | 0.411 |
| 10 | 0.282 | 0.209 | 0.291 | 0.503 | 0.128 | 0.306 |
| 11 | 0.005 | 0.045 | 0.044 | 0.015 | 0.025 | 0.098 |
| 12 | 0.045 | 0.105 | 0.105 | 0.069 | 0.138 | 0.085 |

Table III P-value of Questionnaires and Performance (Scheirer–Ray–Hare Test)

|  | Rest condition effect | Measured time effect | Interaction effect |
| --- | --- | --- | --- |
| Engagement | 0.288 | 0.016 | 0.997 |
| Distress | 0.527 | 0.008 | 0.972 |
| Worry | 0.367 | 0.516 | 0.924 |
| General fatigue | 0.184 | 0.001 | 0.925 |
| Physical fatigue | 0.563 | 0.018 | 0.727 |
| Mental fatigue | 0.833 | 0.000 | 0.548 |
| Reduced activity | 0.531 | 0.802 | 0.794 |
| Reduced motivation | 0.730 | 0.026 | 0.212 |
| PVC | 0.517 | 0.811 | 0.852 |
| ITR | 0.481 | 0.664 | 0.888 |

Table IV Questions of SSSQ and MFI-20

| English | Chinese |
| --- | --- |
| Dissatisfied | 我感到不满意 |
| Alert | 我感到警惕 |
| Depressed | 我感到抑郁 |
| Sad | 我感到忧伤 |
| Active | 我感到活跃 |
| Impatient | 我感到不耐烦 |
| Annoyed | 我感到恼怒 |
| Angry | 我感到生气 |
| Irritated | 我感到被激怒 |
| Grouchy | 我感到不满 |
| I am committed to attaining my performance goals | 我投入地完成今天的目标 |
| I want to succeed on the task | 我今天想要获得成功 |
| I am motivated to do the task | 我今天充满动力 |
| I'm trying to figure myself out | 我在试着理解自己 |
| I'm reflecting about myself. | 我在反思自己 |
| I’m daydreaming about myself. | 我对自己抱有幻想 |
| I feel confident about my abilities. | 我对自己的能力感到自信 |
| I feel self-conscious. | 我有自我意识 |
| I am worried about what other people think of me. | 他人对我的看法让我感到担忧 |
| I feel concerned about the impression I am making. | 我在乎我给他人留下的形象 |
| I expect to perform proficiently on this task. | 我期待今天能够表现的熟练 |
| Generally, I feel in control of things. | 通常而言，我认为事物都在掌控中 |
| I thought about how others have done on this task. | 我考虑过别人今天会做些什么 |
| I thought about how I would feel if I were told how I performed. | 我考虑过如果我被告知今天的表现，我会有怎样的感受 |
| I feel fit. | 我感觉不错 |
| Physically, I feel only able to do a little. | 我感觉我的体力使我只能做少量工作 |
| I feel very active. | 我感觉自己精力充沛 |
| I feel like doing all sorts of nice things. | 我想要做各种自己感觉好的事情 |
| I feel Tired. | 我觉得累 |
| I think I do a lot in a day. | 我认为一天中我做了很多事 |
| When I am doing something, I can keep my thoughts on it. | 我在做事时能够集中注意力 |
| Physically I can take on a lot. | 根据我的身体状况，我能承担很多工作 |
| I dread having to do things. | 我害怕必须做事 |
| I think I do very little in a day. | 我认为我一天中做的事情太少了 |
| I can concentrate well. | 我能够很好地集中注意力 |
| I am rested. | 我休息的不错 |
| It takes a lot of effort to concentrate on things. | 我要集中注意力很费劲 |
| Physically I feel I am in a bad condition. | 我觉得自己的身体状况不好 |
| I have a lot of plans. | 我有很多想做的事情 |
| I tire easily | 我容易疲倦 |
| I get little done. | 我做的事情很少 |
| I don't feel like doing anything. | 我不想做任何事 |
| My thoughts easily wander. | 我的思想很容易走神 |
| Physically I feel I am in an excellent condition. | 我感觉身体状况非常好 |
